# The influence of CRS and ICANS on the efficacy of anti-CD19 CAR-T treatment for B-cell acute lymphoblastic leukemia

**DOI:** 10.3389/fimmu.2024.1448709

**Published:** 2024-09-27

**Authors:** Yuhan Ma, Hongyuan Zhou, Jiaoli Zhang, Qing Zhang, Yujie Li, Ruiyang Xie, Bingpei Zhang, Ziyuan Shen, Ping Li, Aibin Liang, Keshu Zhou, Lu Han, Yongxian Hu, Kailin Xu, Wei Sang, Xiangmin Wang

**Affiliations:** ^1^ Department of Hematology, Suqian First Hospital, Suqian, China; ^2^ Department of Hematology, The Affiliated Hospital of Xuzhou Medical University, Xuzhou, China; ^3^ Blood Diseases Institute, Xuzhou Medical University, Xuzhou, China; ^4^ Department of Rehabilitation, The Affiliated Hospital of Xuzhou Medical University, Xuzhou, China; ^5^ Department of Epidemiology and Biostatistics, School of Public Health, Anhui Medical University, Hefei, Anhui, China; ^6^ Department of Hematology, Tongji Hospital of Tongji University, Shanghai, China; ^7^ Department of Hematology, The Affiliated Cancer Hospital of Zhengzhou University, Zhengzhou, China; ^8^ Department of Hematology, The First Affiliated Hospital, Zhejiang University School of Medicine, Hangzhou, China

**Keywords:** cytokine release syndrome, immune effector cell-associated neurotoxicity syndrome, chimeric antigen receptor T cell therapy, relapsed/refractory B cell lymphoblastic leukemia, efficacy

## Abstract

**Background:**

Chimeric antigen receptor T-cell (CAR-T) therapy has offered new opportunities for patients with relapsed/refractory B-cell lymphoblastic leukemia (r/r B-ALL). However, cytokine release syndrome (CRS) and immune effector cell–associated neurotoxicity syndrome (ICANS) are the two most common toxicities following CAR-T cell therapy. At present, whether the occurrence of CRS and ICANS will impact CAR-T activity remains unknown; this affects the therapeutic efficacy of CAR-T.

**Methods:**

In this multicenter retrospective study, we enrolled 93 patients with r/r B-ALL receiving anti-CD19 CAR-T cell therapy at four medical centers. We evaluated their complete response (CR) rates, minimal residual disease (MRD)-negative CR rates, and survival outcomes.

**Results:**

Among the included patients, 76 (81.7%) developed CRS and 16 (5.3%) developed ICANS. Fifteen patients experienced concurrent CRS and ICANS. However, no significant differences were noted in CR or MRD-negative CR rates between patients with and without CRS/ICANS. Furthermore, no significant difference was noted in leukemia-free survival (LFS) (p = 0.869 for CRS and p = 0.276 for ICANS) or overall survival (OS) (p = 0.677 for CRS and p = 0.326 for ICANS) between patients with and without CRS/ICANS. Similarly, patients with concurrent CRS and ICANS exhibited no differences in OS and LFS when compared with other patients. Multivariate analysis showed that the development of CRS and ICANS was not associated with any difference in OS and LFS.

**Conclusion:**

Patients with CRS/ICANS experience similar clinical outcomes compared with those without CRS/ICANS following anti-CD19 CAR-T therapy.

## Introduction

Chimeric antigen receptor T-cell (CAR-T) therapy has recently emerged as a promising new treatment modality, with a remarkable progress in patients with hematologic malignancies, particularly in those with relapsed/refractory B-cell lymphoblastic leukemia (r/r B-ALL) (1–3). In our previous studies, we reported that CD19-targeting CAR-T therapy exhibits a complete remission (CR) rate of more than 90% in patients with r/r B-ALL (1, 4, 5). However, cytokine release syndrome (CRS) and immune effector cell–associated neurotoxicity syndrome (ICANS) encompass the two most notable toxicities following CAR-T therapy, resulting in treatment challenges in patients.

As an adverse event that occurs in up to 80%–90% of patients receiving CAR-T therapy, CRS is triggered by robust and exponential CAR-T expansion, with severe CRS (grade 3 or higher) having an incidence of 20%–45% (6, 7). CRS occurrence is a double-edged sword. On the one hand, CRS or ICANS occurrence is the hallmark of CAR-T expansion *in vivo* (8–10). Patients who have not experienced any CRS or ICANS may exhibit low levels or even lack CAR-T expansion, thereby resulting in poorer clinical outcomes (9). On the other hand, severe CRS is typically associated with the dysfunction of multiple vital organs, including the heart, liver, and kidneys; this can be fatal and affect the survival benefits of this therapy (11). In our previous study, we noted that severe CRS is associated with shorter progression-free survival (PFS) and overall survival (OS) in patients with multiple myeloma (12). In contrast, two other studies (13, 14) revealed a non-significantly decreased response rate in patients with relapsed/refractory aggressive B-cell non-Hodgkin lymphoma and severe CRS. Furthermore, corticosteroids used in clinical settings to control CRS may affect the efficacy of CAR-T (3, 15, 16). ICANS is another common adverse event following CAR-T therapy; it occurs concomitantly with or after CRS and is observed in 50%–65% of patients (2, 8, 17, 18). At present, the effect of ICANS on efficacy after CAR-T therapy remains unclear. In a cohort study on axicabtagene ciloleucel in large B-cell lymphoma, high-grade ICANS was associated with shorter PFS and OS (19). Collectively, data on the response rate and survival of patients with ALL and concomitant CRS and ICANS after CAR-T treatment is limited at present.

A few risk factors are associated with the efficacy of CAR-T therapy, including the number of CAR-T infusions, tumor burden, and intensity of lymphocyte-depleting chemotherapy (3, 16, 20). However, information on the associations between toxicities (CRS and ICANS) and efficacies following CAR-T therapy is limited. Therefore, in this multicenter retrospective study, we investigated whether the absence of CRS and ICANS reflects the clinical efficacy of patients with ALL receiving CAR-T therapy and whether CRS and ICANS development is associated with improved or worsened survival outcomes.

## Patients and methods

### Patients

The data of patients with CD19+ r/r B-ALL who received anti-CD19 CAR-T therapy between May 2016 and December 2022 at four medical centers were retrospectively reviewed. The enrolled patients had relapsed or refractory disease. The eligibility criteria were as follows: less than 70 years of age; good organ function and survival of more than 3 months; and Eastern Cooperative Oncology Group performance status of <2. Signed informed consent was provided by all patients before anti-CD19 CAR-T therapy. The Ethics Committee of the participating centers approved the study, in compliance with the Helsinki Declaration.

### CAR-T therapy

Using a previously described method, anti-CD19 CAR-T cells constructed with the 4-1BB co-stimulatory domain were generated using a lentiviral vector (1). All anti-CD19 CAR-T underwent quality control before release. All patients received lymphocyte-depleting chemotherapy with a fludarabine and cyclophosphamide regimen (fludarabine at 30 mg/m^2^ per day on days −5 to −3 and cyclophosphamide at 750 mg/m^2^ on day −5). Thereafter, they provided signed informed consent for undergoing anti-CD19 CAR-T therapy and received a median dose of autologous anti-CD19 CAR-T infusion of 1.1 × 10^6^ CAR-T/kg.

### Toxicity assessment

CRS was graded on the basis of the American Society for Transplantation and Cellular Therapy (ASTCT) CRS consensus grading (21). CRS was considered severe if it was grade 3 or higher. ICANS was evaluated on the basis of the ASTCT ICANS consensus grading for neurologic toxicity associated with immune effector cells (21). Severe neurotoxic effects were defined as a seizure of any grade or a toxic effect of grade 3 or higher.

### Response assessment

Morphological analysis and multicolor flow cytometry were performed to assess response to therapy. Complete response (CR) was defined as the presence of less than 5% of bone marrow blasts, the absence of circulating blasts, and no extramedullary disease sites (as assessed via computed tomography or positron emission tomography), regardless of cell count recovery. MRD negativity was defined as the presence of less than 0.01% of bone marrow blasts for samples subjected to multicolor flow cytometry (22). Relapsed disease was defined as the reappearance of blasts in the blood or bone marrow or in an extramedullary site after CR. Quantitative real‐time polymerase chain reaction was performed to measure CAR DNA copies in the peripheral blood (PB) as copies per microgram of genomic DNA. OS was defined as the time from infusion to the date of death from any cause. Leukemia-free survival (LFS) was calculated from the date of CR to the date of relapse, death, or the last follow-up.

### Measurement of serum interleukin-6 and ferritin levels

Enzyme linked immunosorbent assay (ELISA) was performed according to the manufacturer’s instructions (R&D Systems, Minneapolis, USA) to measure serum interleukin-6 (IL-6) and ferritin levels.

### Statistical analyses

Median and range were used to describe all measurement data. Comparisons were made using Mann–Whitney U tests. Frequency (%) was used to express enumeration data, with comparisons being made using chi-squared tests or Fisher’s exact test. The Kaplan–Meier method was utilized to determine follow-up time, OS, and LFS. All tests were two-sided, with a p-value of <0.05 being considered statistically significant. SPSS (version 26.0; IBM, Armonk, NY) and GraphPad Prism 8 software (La Jolla, CA) were used to perform data analyses.

## Results

### Patient characteristics

Ninety-three patients were enrolled in this study, with 47 men and 46 women. The median age was 28 years (range 22–70 years). **Table 1** summarizes the characteristics of the patients. CRS was identified as the most common non-hematological adverse event and occurred in 76 (81.7%) patients, with 19 (20.4%) patients developing severe CRS. The median time for CRS and severe CRS occurrence was day 5 (range: 1–14 and 1–13 days, respectively). Among the enrolled patients, 16 (5.3%) experienced ICANS, with 8 (2.65%) experiencing severe ICANS. The median time for ICANS and severe ICANS occurrence was days 8 and 7 (range: 4–19 and 5–19 days, respectively). Furthermore, 15 (16.1%) patients experienced CRS concomitant ICANS. Among the 93 patients, 51 (54.8%) received corticosteroids for managing CAR-T therapy–associated toxicities. The median cumulative dexamethasone equivalent dose was 115 mg (range: 7.5–956 mg), and the median corticosteroid treatment duration was 5 days (range: 1–20 days). The median time for corticosteroid administration was day 8 (range: 3–14 days). Forty-five (48.3%) patients received tocilizumab. The median cumulative dose of tocilizumab was 480 mg (range: 80–1,280 mg) and the median duration of tociliuzumab treatment was day 1 (range: 1–2). The median time for tocilizumab administration was day 7 (range: 2*–*15 days).

**Table 1:** Patient characteristics.

Characteristics	CRS		p	ICANS		p	CRS concomitant ICANS		p
	Yes (n = 76)	No (n = 17)		Yes (n = 16)	No (n = 77)		Yes (n = 15)	No(n = 78)	
Median age (range)	31 (2–70)	26 (2–66)	0.735	20 (6–68)	32 (2–70)	0.460	19 (6–68)	31 (2–70)	0.344
Gender			0.164			0.962			0.962
Male, n (%)	41 (53.95%)	6 (35.29%)		8 (50.00%)	39 (50.65%)		8 (53.33%)	39 (50.00%)	
Female, n (%)	35 (46.05%)	11 (64.71%)		8 (50.00%)	38 (49.35%)		7 (46.67%)	39 (50.00%)	
Ph, n (%)			0.246			0.946			0.945
Positive	13 (17.11%)	5 (29.41%)		3 (18.75%)	15 (19.48%)		3 (20.00%)	15 (19.23%)	
Negative	63 (82.89%)	12 (70.59%)		13 (81.25%)	62 (80.52%)		12 (80.00%)	63 (80.76%)	
Peak levels of IL-6			<0.001			0.173			0.072
Median (range)	517 (3.1–27231)	8.9 (2.4–481)		94 (7.6–10952.9	255 (2.4–27231)		5,000 (12–10,952.9)	261 (6–5,000)	
Peak levels of ferritin			0.141			0.248			0.248
Median (range)	2,781 (153–76,301)	1,459.5 (106–67,385		9,987 (788–45,323	2,000 (106–76,301)		9,987 (788–45,323)	2,000 (106–76,301)	
Prior therapies lines			0.510			0.019			0.046
Median (range)	3.5 (1–13)	4 (0–7)		2 (1–6)	4 (0–13)		2 (1–6)	4 (1–13)	
Disease Status			0.704			0.443			0.371
Primary refractory, n (%)	9 (11.84%)	3 (17.65%)		3 (18.75%)	9 (11.69%)		3 (20.00%)	9 (11.54%)	
Relapsed, n (%)	67 (88.16%)	14 (82.35%)		13 (81.25%)	68 (88.31%)		12 (80.00%)	69 (88.46%)	
Number of relapses			0.056			0.063			0.063
Median (range)	1 (1–4)	2 (1–4)		1 (0–2)	1 (0–4)		1 (1–2)	1 (1–4)	
Received steroids			<0.001			0.002			0.007
Yes, n (%)	51 (67.11%)	0 (0%)		13 (81.25%)	38 (49.35%)		13 (86.67%)	38 (48.72%)	
No, n (%)	25 (32.89%)	17 (100%)		3 (18.75%)	39 (50.65%)		2 (13.33%)	40 (51.28%)	
Received tocilizumab			<0.001			0.019			0.007
Yes, n (%)	45 (59.21%)	0 (0%)		12 (75.00%)	33 (42.86%)		12 (80.00%)	33 (42.31%)	
No, n (%)	31 (40.79%)	17 (100%)		4 (25.00%)	44 (57.14%)		3 (20.00%)	45 (57.69%)	
BM blasts before CAR-T			0.445			0.191			0.098
Median (range)%	23.75 (0–92)	5.15 (0–95)		55 (0–80)	33 (0–95)		62 (8–80)	33 (0–95)	
Infused cells			0.025			0.420			0.513
Median (range) * 10^6^/kg	1.2 (0.4–4.3)	1.0 (0.5–3)		1 (0.9–4)	1 (0.4–4.3)		1 (1–4)	1 (0.4–2)	

CRS, severe cytokine release syndrome; ICANS, immune effector cell–associated neurotoxicity BM, bone marrow; CAR-T, chimeric antigen receptor T-cell (CAR-T) therapy.

The symbol *means multiplication.

CRS was associated with peak IL-6 levels throughout the treatment period; however, no statistically significant difference was noted in IL-6 peak levels between patients with and without ICANS, patients with CRS concomitant ICANS, and others. Patients who received higher doses of CAR-T infusion had a higher probability of developing CRS (p < 0.025). Nevertheless, no significant differences were noted in the incidence of CRS, ICANS, and CRS concomitant ICANS based on sex, presence of Ph chromosome, peak ferritin levels, prior therapy lines, disease status, number of relapses, and number of BM blasts before CAR-T therapy.

### Clinical response rate analysis

**Figure 1:**
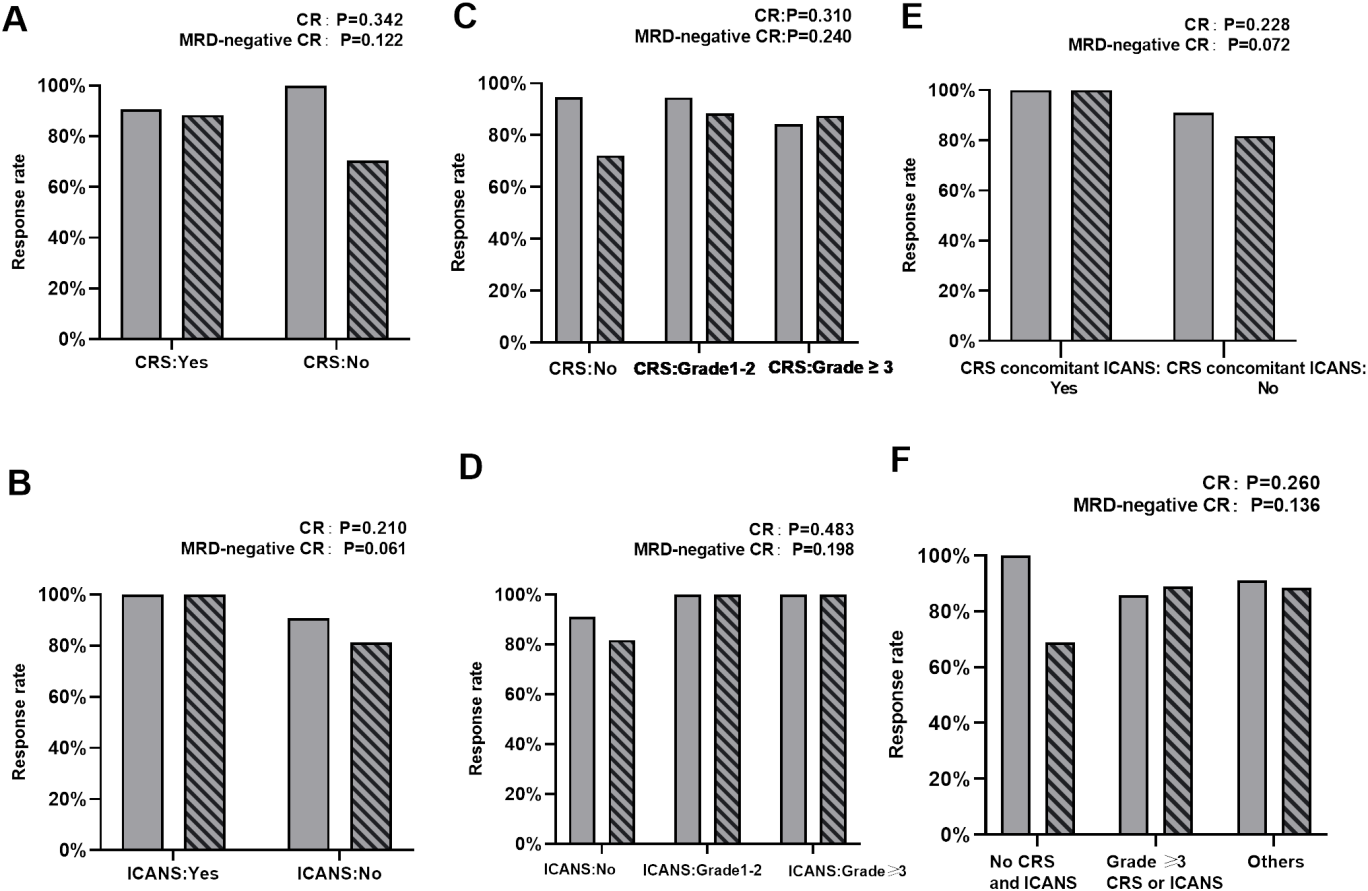
Comparison of CR and MRD‐negative CR rates. **(A)** Comparison of CR and MRD‐negative CR rates between patients with and without CRS. **(B)** Comparison of CR and MRD‐negative CR rates between patients with and without ICANS. **(C)** Comparison of CR and MRD‐negative CR rates between patients with no CRS, grade 1–2 CRS, and ≥ grade 3 CRS. **(D)** Comparison of CR and MRD‐negative CR rates between patients with no ICANS, grade 1–2 ICANS, and ≥ grade 3 ICANS. **(E)** Comparison of CR and MRD‐negative CR rates between patients who develop concurrent CRS and ICANS or not. **(F)** Comparison of CR and MRD‐negative CR rates between patients without CRS and ICANS, ≥grade 3 CRS or ICANS, and other patients.

Among the 93 patients who received anti-CD19 CAR-T infusion, 86 (92.5%) achieved CR at day 30. The response rates were evaluated in patients with or without CRS/ICANS and are summarized in **Figure 1**. The CR and MRD-negative CR rates were similar between patients with and without CRS (p = 0.342 and 0.122, respectively). Similar results were obtained in patients with or without ICANS (p = 0.210 and 0.061, respectively) and CRS concomitant ICANS and others (p = 0.228 and 0.072, respectively). When stratified on the basis of the severity of CRS/ICANS, no difference was noted in CR and MRD-negative CR rates among patients without CRS/ICANS, grade 1–2 CRS/ICANS, and grade 3 or higher CRS/ICANS. Compared with patients without CRS and ICANS, other patients (grade 1–2 CRS and/or ICANS, whether occurring together or alone) and patients with grade 3 or higher CRS or ICANS yield similar CR and MRD-negative CR rates (p = 0.260 and 0.136, respectively). Furthermore, no significant differences were noted between the two groups who did or did not receive tocilizumab in terms of CR rates (p = 1). Corticosteroids were also reserved for managing severe CRS and ICANS and did not appear to affect CR rates (p = 0.697).

### Survival outcomes

**Figure 2:**
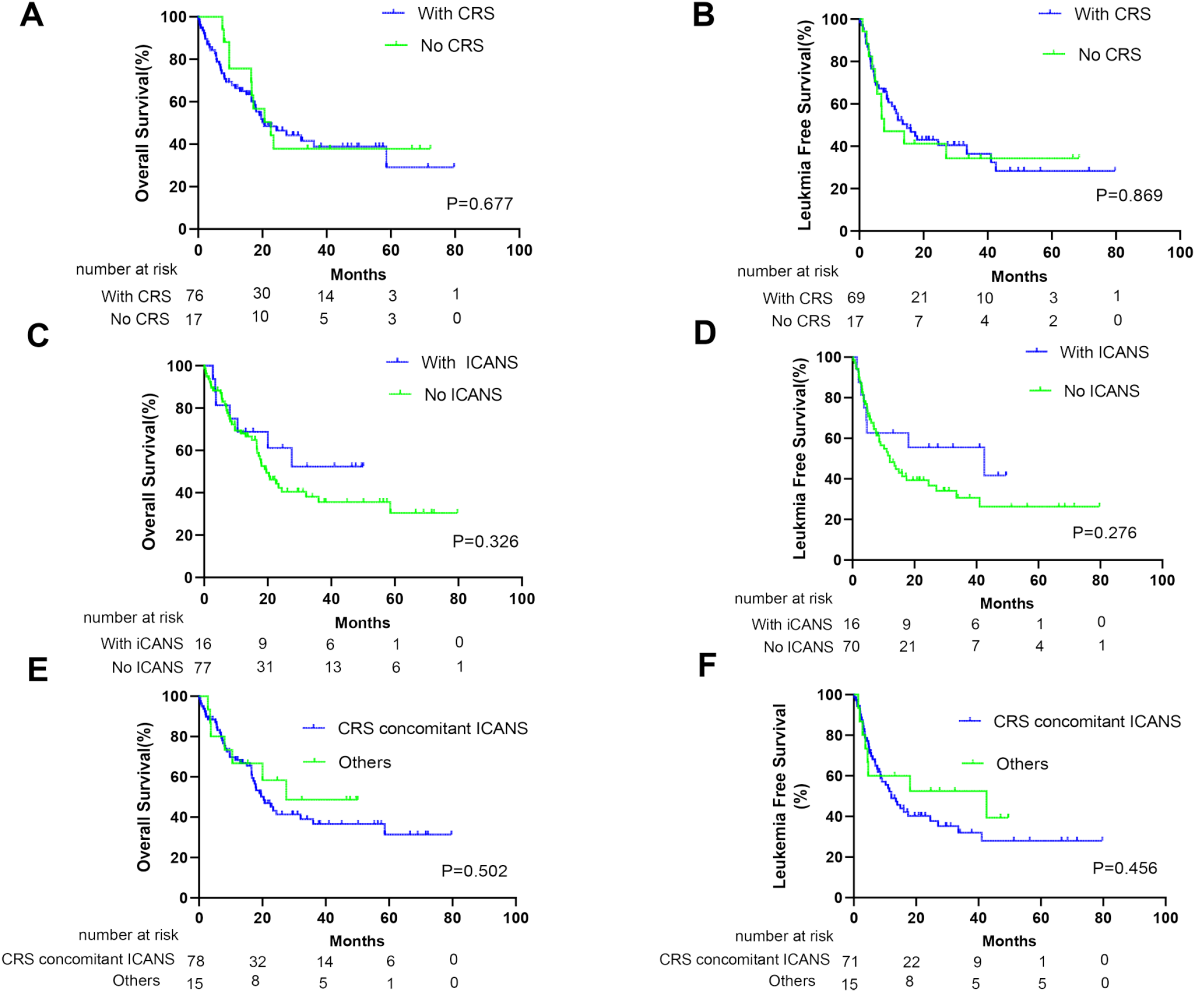
Prognosis of patients after anti-CD19 CAR-T therapy according to CRS and ICANS. The overall survival (OS) of all patients **(A)** and leukemia-free survival (LFS) of CR patients **(B)** with or without CRS. The OS of all patients **(C)** and LFS of CR patients **(D)** with or without ICANS. The OS of all patients **(E)** and LFS of CR patients **(F)** who develop concurrent CRS and ICANS or not.

**Figure 3:**
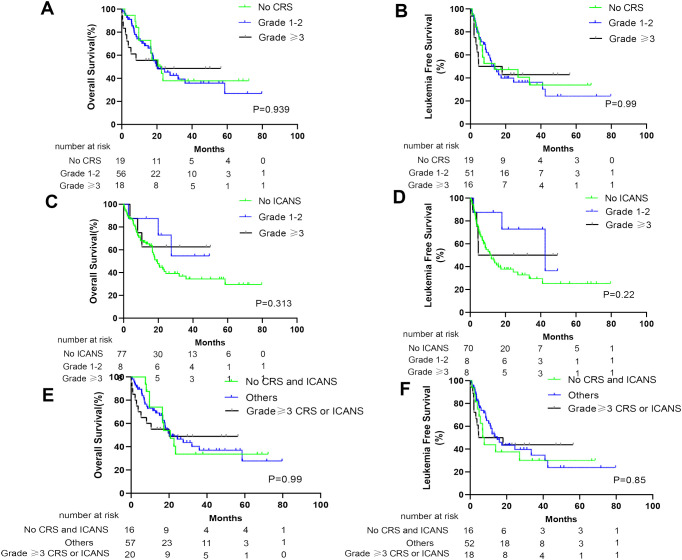
Prognosis of patients after anti-CD19 CAR-T therapy stratified by degree of CRS and ICANS. The OS of all patients **(A)** and LFS of CR patients **(B)** with no CRS, grade 1–2 CRS, and ≥ grade 3 CRS. The OS of all patients **(C)** and LFS of CR patients **(D)** with no ICANS, grade 1–2 ICANS, and ≥ grade 3 ICANS. The OS of all patients **(E)** and LFS of CR patients **(F)** without CRS and ICANS, ≥grade 3 CRS or ICANS, and other patients.

At the cutoff date (1 March 2023), the median follow-up time was 38.3 months (95% CI, 26.6–50.0 months). No significant difference was noted in OS and LFS between patients with and without CRS (p*=* 0.677 and 0.869, respectively; **Figures 2A, B**). Furthermore, similar results were obtained when comparing patients with and without ICANS (p *=* 0.326 and 0.276, respectively; **Figures 2C, D**). In addition, patients with CRS concomitant ICANS did not exhibit significant differences in OS and LFS when compared with other patients (p = 0.502 and 0.456, respectively; **Figures 2E, F**). Stratified analysis of CRS grading revealed no differences in the survival outcomes of patients who did not develop CRS, those who developed grade 1–2 CRS, and those who developed ≥grade 3 CRS (**Figures 3A, B**). Similar results were obtained in the stratified analysis of ICANS grading in patients with ALL (**Figures 3C, D**). Lastly, no significant differences were noted in OS and LFS in patients without CRS and ICANS, those with ≥grade 3 CRS or ICANS, and others (p = 0.99 and 0.85, respectively; **Figures 3E, F**). Compared with the non-tocilizumab group, the OS and LFS of the tocilizumab group were not significantly different (P = 0.773 for OS and 0.985 for LFS). Compared with patients who did not receive corticosteroids, the use of corticosteroids did not affect the OS and LFS (p = 0.099 for OS and p = 0.176 for LFS).

### Anti‐CD19 CAR-T kinetics

**Figure 4:**
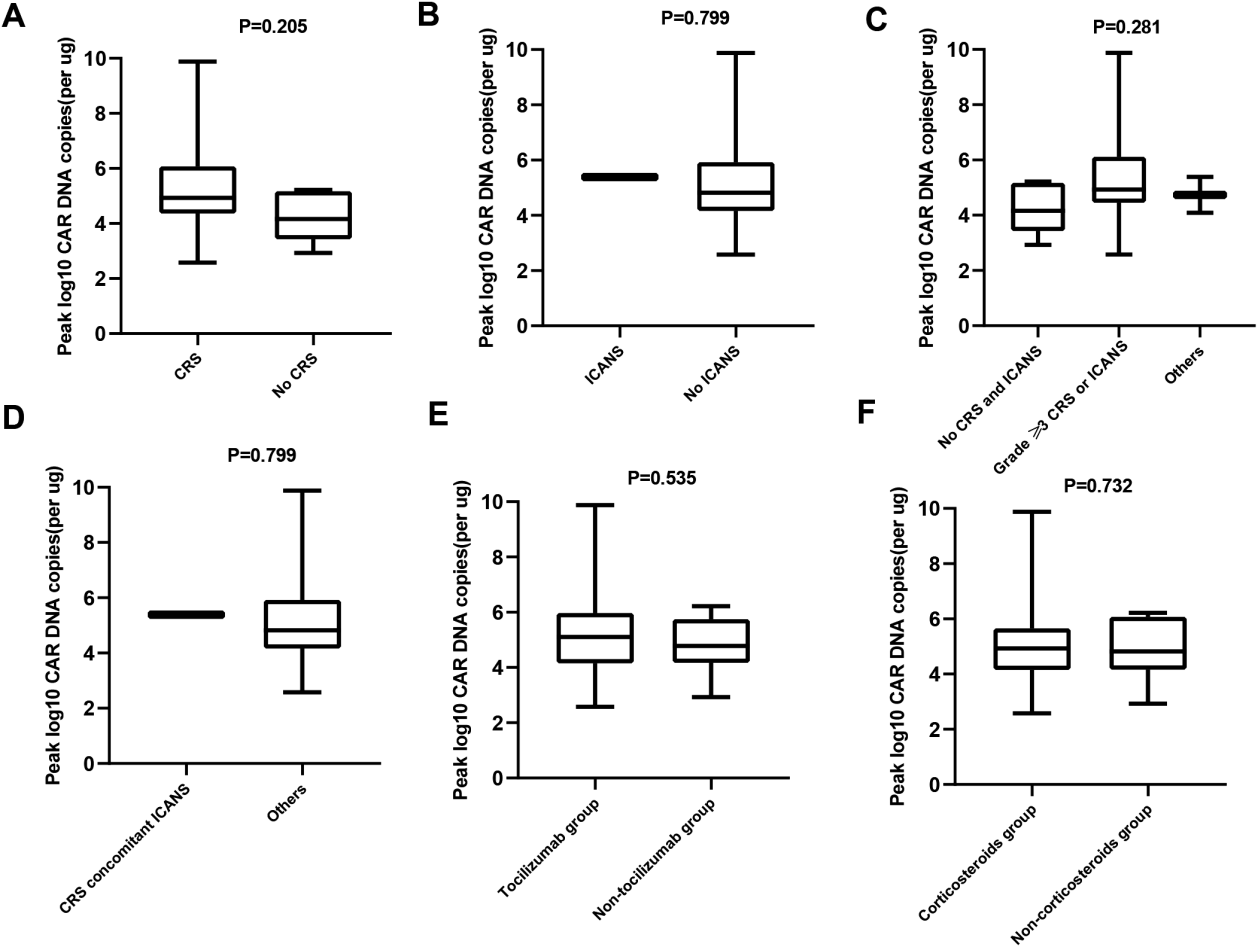
Expansion of CAR T cells in the peripheral blood (PB) of patients assessed by qPCR. **(A)** Comparison of peak CAR-T cell expansion in the PB of patients with or without CRS. **(B)** Comparison of peak CAR-T cell expansion in the PB of patients with or without ICANS.**(C)** Comparison of peak CAR-T cell expansion in the PB of patients without CRS/ICANS, ≥grade 3 CRS/ ICANS, and others. **(D)** Comparison of peak CAR-T cell expansion in the PB of patients with CRS concomitant ICANS and others. **(E)** Comparison of peak CAR-T cell expansion in the PB of patients receiving tocilizumab or not **(F)** Comparison of peak CAR-T cell expansion in the PB of patients receiving corticosteroids or not.

The expansion and persistence of CAR-T were evaluated in 24 patients with sufficient PB specimens for analysis (**Figure 4**). Patients with and without CRS had comparable median peak numbers of CAR DNA copies in the PB (p = 0.205; **Figure 4A**). Similar results were observed in patients with or without ICANS (p = 0.799; **Figure 4B**), patients without CRS/ICANS, those with ≥grade 3 CRS/ICANS, and others (p = 0.281; **Figure 4C**), as well as patients with CRS concomitant ICANS and others (p = 0.799; **Figure 4D**). Compared with the non-tocilizumab group, there was no effect on CAR-T expansion in the tocilizumab group (p = 0.535; **Figure 4E**). Similar results were observed in patients who did or did not receive steroids (p = 0.732; **Figure 4F**).

### Multivariate analysis

Univariate analysis revealed that age, sex, prior therapy lines, and number of infused cells did not significantly affect the OS and LFS of patients. Candidate variables that were considered clinically significant or had a p-value of <0.1 in univariate analysis were included in the multivariate Cox proportional hazards regression model. Multivariate analysis revealed that the development of ≥grade 3 CRS or ICANS was not associated with a difference in OS and LFS, with hazard ratios (HRs) of 2.021 (95% CI: 0.653–6.256, p = 0.222) and 0.785 (95% CI: 0.250–2.466, p = 0.678), respectively. Similarly, other factors (development of grade 1–2 CRS and/or ICANS, whether occurring simultaneously or separately) were not significantly associated with differences in OS and LFS, with HRs of 1.379 (95% CI: 0.611–3.116, p = 0.439) and 0.788 (95% CI: 0.342–1.815, p = 0.576), respectively. Furthermore, no significant differences were noted in LFS or OS based on the number of BM blasts (≥20% vs. <20%). Compared with patients who did not receive these treatments, no significant effect was noted on LFS or OS in patients who received steroids or tocilizumab during the treatment (**Table 2**).

**Table 2:** Multivariate Cox regression analysis for OS and LFS of CR patients.

Subgroup	HR	95% CI	P
OS			
BM blasts^#^ ≥20% vs. <20%	0.890	0.485–1.633	0.707
Received steroids	0.547	0.271–1.103	0.092
Received tocilizumab	1.031	0.512–2.076	0.932
Group			0.468
No CRS and ICANS	Reference		
Grade ≥3 CRS or ICANS	2.021	0.653–6.256	0.222
Others	1.379	0.611–3.116	0.439
LFS			
BM blasts^#^ ≥20% vs. <20%	1.441	0.761–2.732	0.262
Received steroids	0.698	0.334–1.458	0.338
Received tocilizumab	1.207	0.561–2.598	0.630
Group			
No CRS and ICANS	Reference		0.855
Grade ≥3 CRS or ICANS	0.785	0.250–2.466	0.678
Others	0.788	0.342–1.815	0.576

OS, overall survival; LFS, leukemia-free survival; BM, bone marrow; ^#^ before infusion.

## Discussion

CAR-T therapy has exhibited high efficacy against r/r B-cell malignancies, with particularly high response rates in patients with B-ALL. However, various toxicities, such as CRS and ICANS, remain significant barriers to the widespread dissemination of this promising therapy. Some studies have revealed that these toxicities not only affect the response rates of CAR-T but also affect patient survival, with the final outcome being associated with the severity of toxicities (23–25). However, researchers have not raised concerns about the lack of response to CAR-T therapy in patients with r/r ALL who have not experienced CRS or ICANS. In this retrospective study, we investigated whether these toxicities affect the efficacy of CAR-T therapy. We noted that CRS and ICANS do not affect the response rate and long-term survival of patients with R/R ALL receiving CAR-T therapy.

CRS typically presents with symptoms of fever, hypotension, and hypoxia. It initially represents excessive immune responses and is frequently used informally as a surrogate marker for CAR-T activity. This may indicate that CAR-T exerts therapeutic effects. Some degree of CRS may achieve an effective response to therapy (26). However, severe CRS is a cytokine storm, resulting in significant damage to tissues and organs, possibly affecting the efficacy of CAR-T. Therefore, whether patients with severe CRS have inferior response rates or survival remains controversial (12–14). Our efficacy study findings suggest no differences in CR and MRD-negative CR rates between patients with and without CRS. This finding was consistent when comparing patients without CRS, those with grade 1–2 CRS, and those with grade ≥3 CRS (**Figures 1A, B**). At present, many studies have focused on the risk factors for prognosis following CAR-T therapy in patients (27–31). However, few studies have revealed whether CRS severity affects long-term survival. In our study, CRS was not associated with a difference in survival (**Figures 2A, B**). When further stratified on the basis of CRS grade, it remained consistent when comparing the OS and LFS of patients with grade 1–2 CRS, those with ≥grade 3 CRS, and those without CRS. These results are consistent with those of previous studies (13, 14, 32) and further support the idea that clinical CRS is not warranted for the efficacy of CAR-T therapy.

ICANS is another commonly encountered toxicity that clinically manifests as epilepsy, delirium, encephalopathy, dysphasia, tremors, ataxia, dysmetria, aphasia, and confusion. Unlike CRS, the pathophysiology of ICANS remains poorly understood. Only a few studies have focused on whether ICANS development will affect the efficacies of CAR-T therapy and whether severe ICANS will differ from mild ICANS in terms of efficacy. In the present study, similar to the effect of CRS on the efficacy of CAR-T therapy, no statistically significant response rates and survival results were noted for patients who did or did not develop ICANS (**Figures 1B**, **2C**). Furthermore, no differences were observed when stratified based on ICANS severity (**Figures 1D**, **2D**). Most (15/16) patients with ICANS had concurrent CRS, with most cases occurring after CRS onset; this suggests a potential mechanistic relevance. Compared with other patients, no significant differences were noted in the response rate and survival of patients with concurrent CRS and ICANS (**Figures 1E**, **2E, F**).

IL-6 serves as a key mediator of cytokine responses in CRS. We observed a significant correlation between peak IL-6 levels and tumor burden. In particular, the group with ≥20% blast cells had higher IL-6 levels (p < 0.001). This finding is consistent with that of Davila et al. (3). This may explain why patients with a high tumor burden are more likely to develop severe CRS. Tocilizumab, an IL-6 receptor antibody, is typically used as the first-line treatment for CRS, with corticosteroids being reserved for tocilizumab-resistant cases or in combination with ICANS. Unlike CRS, ICANS is characterized by the predominant release of IL-1 from activated mononuclear macrophages rather than IL-6. Subsequently, corticosteroids, and not tocilizumab, are clinically recommended as the first-line treatment for ICANS. Some studies have suggested that glucocorticoids may inhibit the expansion and persistence of CAR-T and affect clinical outcomes (3, 15, 33). However, at present, more studies do not universally support this viewpoint, with some studies even revealing contradictory results (34). In the present study, we also noted that the administration of steroids/tocilizumab was not associated with the CR rate and LFS/OS of patients. The fact that using corticosteroids to manage CRS during CAR-T therapy does not affect patient survival outcomes confers stronger confidence in the practice of therapeutically or prophylactically using steroids in high-risk patients.

In the present study, CRS and ICANS were not associated with clinical outcomes in patients, similar to previous findings (13, 14). The possible reasons why CRS and ICANS do not affect the efficacy of CAR-T therapy and patient survival may be as follows: (1) the expansion and persistence of CAR-T do not differ in patients with or without CRS, those with or without ICANS, those without CRS/ICANS, those with grade ≥3 CRS/ICANS, and others. (2) Tocilizumab or steroids used for toxicity control did not affect the expansion and efficacy of CAR-T. (3) The relatively young age of the patients may partially offset the effect of CRS/ICANS on their vital organs and even survival. In this study, we demonstrated that the occurrence of CRS and ICANS and the different levels of occurrence do not affect the short-term response and long-term survival of patients with r/r B-ALL. This finding helps better understand the factors affecting the efficacies of patients undergoing CAR-T therapy. Considering the small sample size of our retrospective study, more large-scale clinical studies are warranted in the future to further confirm the effect of CRS and ICANS on the efficacy of patients with r/r ALL.

## Data Availability

The original contributions presented in the study are included in the article/supplementary material. Further inquiries can be directed to the corresponding authors.
